# A novel technique for socket-shield preparation used in conjunction with immediate implant placement. A case report

**DOI:** 10.1093/jscr/rjad464

**Published:** 2023-08-14

**Authors:** Cezar Lahham, Elias Edward Lahham, Ali Abu Ali

**Affiliations:** Department of Periodontology, Arab American University, Ramallah, Palestine; Research Department, Al-Quds University, Jerusalem, Palestine; Research Department, Al-Quds University, Jerusalem, Palestine

## Abstract

The socket-shield technique is considered one of the best treatment modalities that reduce the amount of facial bone loss and gingival recession following tooth extraction. However, the difficulty in preparing the shield by partial tooth extraction makes it technique-sensitive and limits its use. This case report presents a 29-year-old medically fit male patient with a destructed non-vital lateral incisor. It was planned for immediate implant placement in conjunction with the socket-shield technique. The shield was prepared in a new technique using a nickel–titanium endodontic file, which was repeatedly adjusted to increase the diameter of the canal gradually. The canal was enlarged to reach a sufficient diameter to perform root separation safely. Following the shield preparation, immediate implant placement was preformed, and screw-retained temporary crown was done.

## INTRODUCTION

Alveolar bone resorption is a common consequence following tooth extraction, that can limit implantological rehabilitation strategies, especially in the maxillary esthetic zone [1]. Many protocols have been used to minimize the negative effect of tooth loss, such as immediate implant placement and socket preservation using bioabsorbable membranes [2]. In 2010, Hurzeler *et al*. has introduced the socket-shield technique (SST), which involves intentional partial retention of the buccal root part, with an immediate implant placed palatal to the retained part, resulting in a predictable, high functional and esthetic outcome [3]. In a systematic review, it has been shown that the SST is characterized by a high survival rate (>98%) and a low complication rate (<4%) [4]. However, SST is considered a sensitive technique, as it is difficult to prepare the shield [5]. Therefore, this case report aims to present a novel, simple and easy technique to prepare the shield properly.

## CLINICAL PRESENTATION

**Figure 1:**
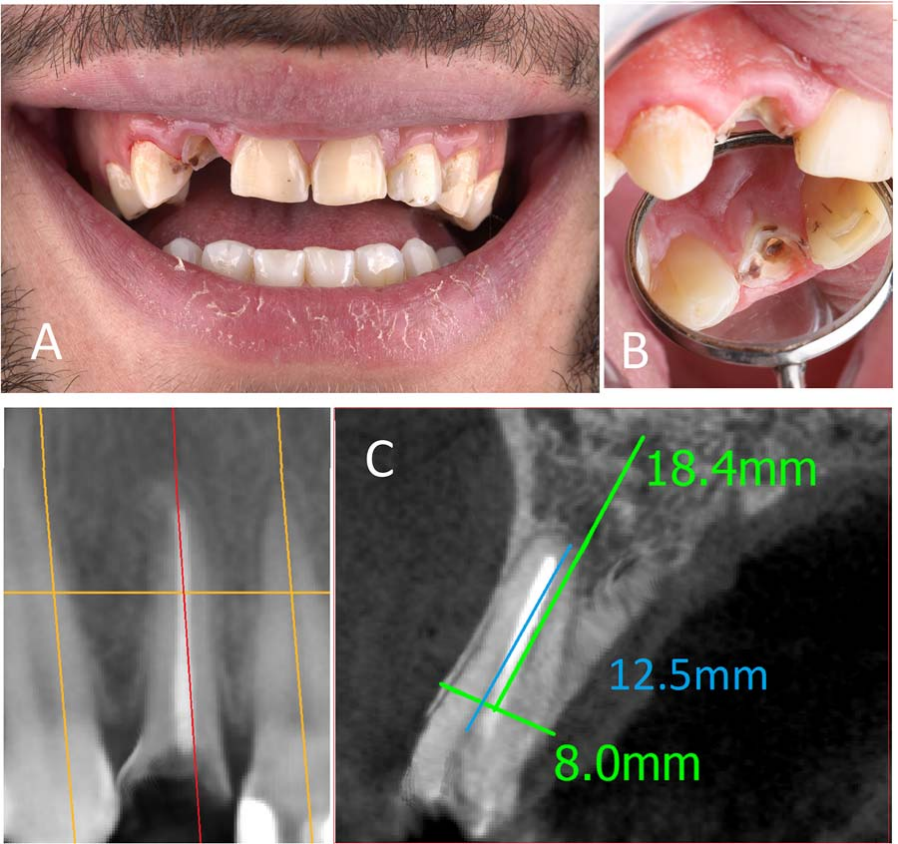
(**A**) Preoperative frontal view, (**B**) preoperative occlusal view, (**C**) preoperative CBCT.

A 29-year-old male patient was referred to the Dental Care Center to replace a fractured upper right lateral incisor (#12) (Fig. 1A and B). The patient was medically fit, non-smoker and with acceptable oral hygiene. During clinical examination, the tooth (#12) was root canal treated, non-mobile, non-tender, had no periodontal pockets >3 mm, had a thin gingival biotype, the gingival margin was at the level of the upper lip line and the tooth was in a good position within the occlusion.

Following that, cone-beam computed tomography (CBCT) was done for bone assessment. There was no periapical lesion, a relatively straight root, and the alveolar crest was at a normal level. However, the buccal plate was very thin (<1 mm) (Fig. 1C).

After a careful clinical and radiological assessment, it was planned to perform an immediate implant placement in conjunction with the SST to reduce the risk of buccal plate fracture during the whole tooth extraction and to maximize the esthetic outcomes.

The procedure was clearly explained to the patient, followed by obtaining an informed consent.

## IMPLANT DIMENSIONS SELECTION

Implant dimensions were determined according to the bucco-palatal, mesio-distal and the apico-coronal distance of the alveolar bone. The bucco-palatal bone dimension in the region of tooth #12 was 8 mm, and the mesio-distal dimension at the same tooth was 6.8 mm. Therefore, the selected implant diameter was 3.75 mm to leave a sufficient bone between the implant and its adjacent. Moreover, when the immediate implant placement is planned, it is recommended to leave 2–3 mm between the buccal implant surface and the buccal bone plate to reduce the risk of dehiscence [6].

For implant length, it should be 3 mm longer than the socket to obtain sufficient primary stability. In this case, the distance between the alveolar crest and the root apex was 12.5 mm, so an implant with 16 mm length was needed.

## SURGICAL PROCEDURE

**Figure 2:**
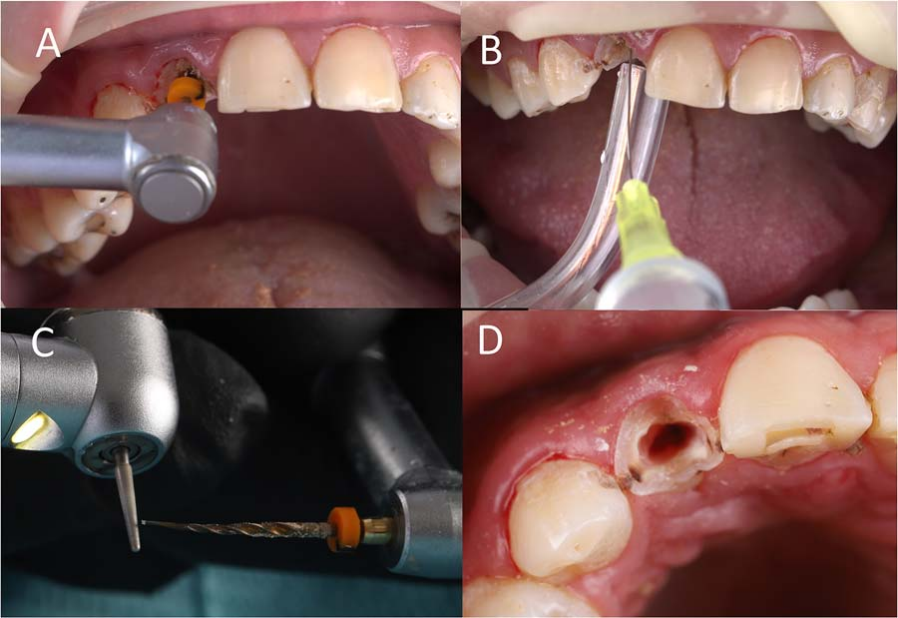
(**A**) Canal preparation, (**B**) canal irrigation, (**C**) file modification, (**D**) canal enlargement.

**Figure 3:**
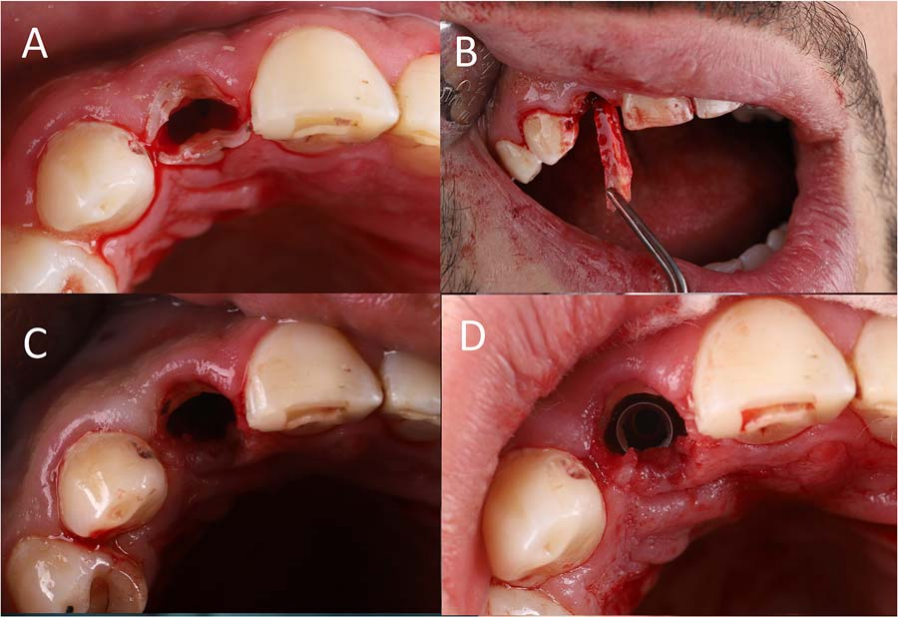
(**A**) Tooth separation, (**B**) partial tooth extraction, (**C**) shield preparation, (**D**) implant placement.

Root canal length was preassessed from the CBCT, and it was 18 mm. A D-finder endodontic file (#8) was used to remove the gutta percha. Then, the canal length was confirmed using the apex locator (it was 17 mm). After canal enlargement to reach file #15, a nickel–titanium (NiTi) rotary file (length: 25 mm, size: 15, taper: 0.06) was used to prepare the canal, reaching the whole canal length (speed: 200 rpm, torque: 3 N.cm) (Fig. 2A). Normal saline was used to remove the debris and to cool down the tooth during preparation (Fig. 2B). Then, the file was trimmed 1 mm from its tip to increase the diameter (Fig. 2C). The stopper location was corrected again, and the preparation of the canal was continued (Fig. 2D). After massive canal enlargement, a long shank tapered diamond bur was used to separate the root mesiodistally. As the patient was not anesthetized, he felt some discomfort when the bur was near the periodontal ligament space. In this step, 2% Lidocaine 1:100 000 was administered buccally and palatally. Complete separation was performed (Fig. 3A). The palatal root section was luxated carefully, and then extracted without any damage to the buccal root section (Fig. 3B).

The buccal section was reduced to just below the base of the gingival sulcus. The shield thickness was also reduced to 1.5 mm. Then, it was beveled and finished using a large diamond round bur (Fig. 3C).

Following shield preparation, an osteotomy was performed palatally to the shield in the cingulum area of the partially extracted tooth (speed 800 rpm, torque 40 N.cm). The implant bed was enlarged to reach a diameter of 3 mm. Then, the implant was inserted 1 mm subcrustal (from the palatal alveolar crest), about 1.5 mm below the shield [7], leaving a 1 mm gap between the implant and the shield (Fig. 3D). The insertion torque was 35 N.cm, which was sufficient for immediate loading also.

## TEMPORARY SCREW-RETAINED CROWN FABRICATION

**Figure 4:**
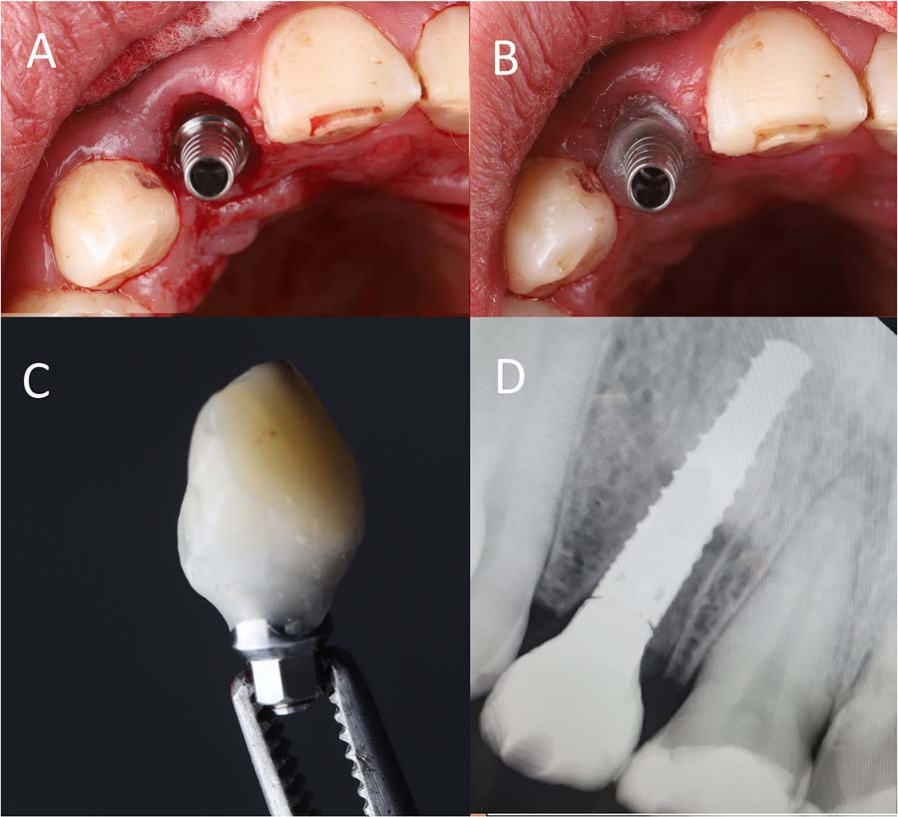
(**A**) Abutment placement, (**B**) adding flowable composite around the abutment, (**C**) crown build-up (**D**) postoperative radiograph.

The implant abutment was placed, and flowable composite was used to fill the gap between the abutment and the gingiva and cured (Fig. 4A).

Then, packable composite was used to build up the temporary crown over the flowable composite (Fig. 4B).

The temporary crown was removed and finished to be concave subgingival to give a space for soft tissue, convex supragingival for producing an emergence profile and out-of-occlusion. Then it was polished properly (Fig. 4C). Finally, periapical radiograph was done immediately postoperative (Fig. 4D).

**Figure 5:**
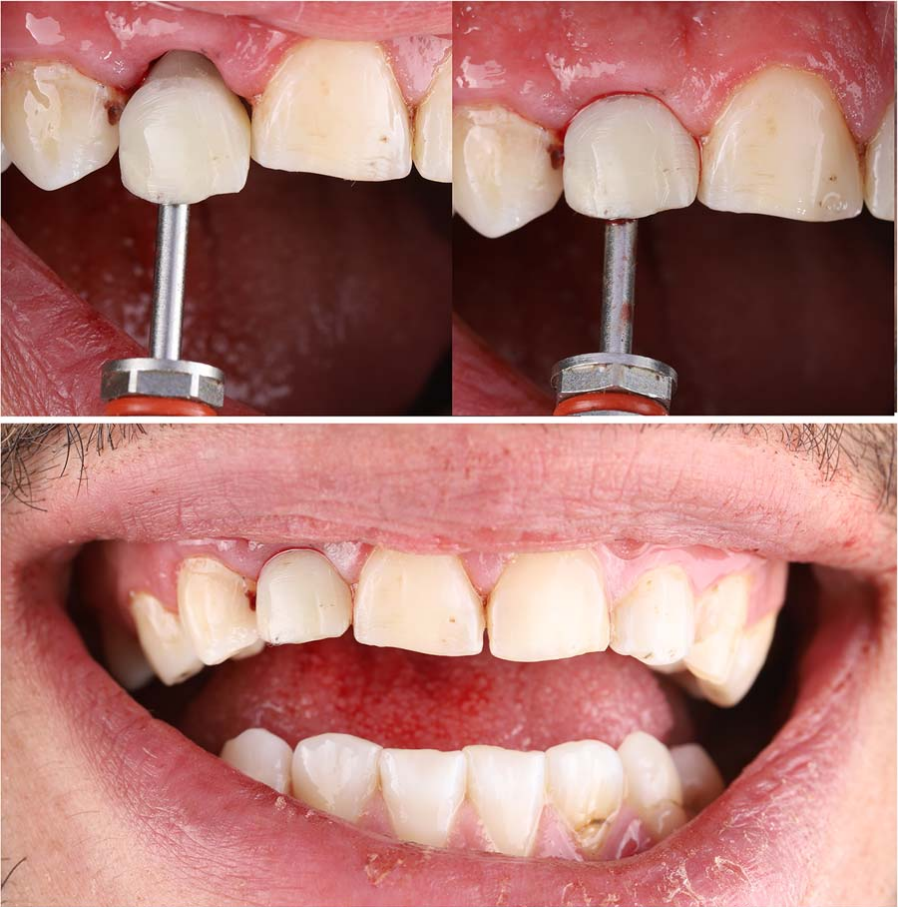
Immediately postoperative clinical photograph.

Patient was instructed to avoid any trauma on this tooth for 3 months, to take Amoxicillin 750 mg twice daily for 1 week and he left the clinic very satisfied (Fig. 5).

## DISCUSSION

Tooth loss in the esthetic zone is typically unpleasant to the patient. A dental implant is the most predictable and cost-effective way to replace lost teeth with a high average life expectancy. However, in the esthetic zone, this procedure is challengeable and may result in some tissue alterations, especially when the delayed implant placement is considered [8].

The SST with immediate implant placement and immediate loading implants has been documented by many authors, showing its role in reducing bone resorption and changes in soft tissues [9], [10]. In a systematic review, it was concluded that the SST maintains buccal soft and hard tissues, which improve the esthetic outcomes compared with other treatment modalities [11]. In this case report, a novel technique for shield preparation was used. Using NiTi files is considered safer and more controllable than using stiff long shank diamond burs during tooth separation.

## CONCLUSION

Within the limitations of this study, it is recommended to use NiTi endodontic files in the initial shield preparation to decrease the risk of improper tooth separation.

## CONFLICT OF INTEREST STATEMENT

None declared.

## FUNDING

None.

## PATIENT CONSENT STATEMENT

Written informed consent was obtained from the patient for the publication of this case report and accompanying images. A copy of the written consent is available for review by the editor-in-chief of this journal on request.

## DATA AVAILABILITY

The data used to support the findings of this study are included within the article.
